# From Engrams to Pathologies of the Brain

**DOI:** 10.3389/fncir.2017.00023

**Published:** 2017-04-07

**Authors:** Christine A. Denny, Evan Lebois, Steve Ramirez

**Affiliations:** ^1^Department of Psychiatry, Columbia UniversityNew York, NY, USA; ^2^Division of Integrative Neuroscience, New York State Psychiatric Institute (NYSPI)/Research Foundation for Mental Hygiene, Inc. (RFMH)New York, NY, USA; ^3^Neuroscience and Pain Research Unit, Pfizer Inc.Cambridge, MA, USA; ^4^Center for Brain Science, Harvard UniversityCambridge, MA, USA

**Keywords:** memory, hippocampus, engram, amygdala, optogenetics, psychiatric disorders, circuits, behavior

## Abstract

Memories are the experiential threads that tie our past to the present. The biological realization of a memory is termed an engram—the enduring biochemical and physiological processes that enable learning and retrieval. The past decade has witnessed an explosion of engram research that suggests we are closing in on boundary conditions for what qualifies as the physical manifestation of memory. In this review, we provide a brief history of engram research, followed by an overview of the many rodent models available to probe memory with intersectional strategies that have yielded unprecedented spatial and temporal resolution over defined sets of cells. We then discuss the limitations and controversies surrounding engram research and subsequently attempt to reconcile many of these views both with data and by proposing a conceptual shift in the strategies utilized to study memory. We finally bridge this literature with human memory research and disorders of the brain and end by providing an experimental blueprint for future engram studies in mammals. Collectively, we believe that we are in an era of neuroscience where engram research has transitioned from ephemeral and philosophical concepts to provisional, tractable, experimental frameworks for studying the cellular, circuit and behavioral manifestations of memory.

## Introduction

We begin with a disclaimer on our interpretation of the state of engram research: we do not know what an engram is fully; we have not found an engram in its entirety; we do not have a complete understanding of the biochemical and physiological parameters underlying engram storage, retrieval and updating. Yet, we continue to use this loaded term in our studies. Why? Because we believe that the data collected so far in studies claiming to have causal leverage over a component of an engram are convincing, robust, reproducible and satisfy many of the criteria surrounding our modern understanding of what qualifies as a memory. As a community, however, we must embrace that the term engram—like any scientific paradigm or framework still in its infancy—is provisional and subject to change as our understanding of memory improves. This is a good thing: given the early stages of engram research, we welcome the opportunity to falsify, revise and update many of the claims regarding engrams because that necessary process of hypothesis testing is the process of science itself. Moreover, we argue that it would make little difference if we performed linguistic gymnastics to avoid the term engram and instead called it a trace, a representation, a memory, Sheena, or a defined neuronal ensemble active during learning that is necessary and sufficient for the behavioral expression of threat detection. The term itself acts as a conceptual pivot point around which experiments can be developed. In short, our goal is to discover and manipulate the underlying neuronal landscape supporting engrams, while heeding semantic, ontological and philosophical caveats. In this review, we provide a conceptual and experimental scaffold for future engram research and to clarify and reconcile many of the recent controversies surrounding a slew of studies claiming to have leverage over such mnemonic processes. While our focus is the mammalian brain and episodic memory, readers are referred elsewhere for excellent reviews focusing on invertebrates (e.g., *C. elegans, Drosophila melanogaster*) or other types of memories (e.g., motor, perceptual; Martin et al., 2000; Horn et al., 2001; Christian and Thompson, 2003; Weinberger, 2004).

## A Century of Memory Research

It’s been almost 100 years since the German zoologist Richard Semon proposed a conceptualization of memory and coined the term engram (Semon, 1921). Semon proposed that an engram is the physical substrate of memory—an enduring change in the brain that results from a particular experience and whose underlying physical substrate can remain dormant until the appropriate external and/or internal cues result in its direct reactivation, thereby leading to retrieval. Decades later, Karl Lashley attempted to localize engrams in the brain by systematically lesioning various functionally connected areas while rodents performed visual discrimination and maze learning tasks (Lashley, 1950). After failing to identify the locus of memory, Lashley wryly concluded, “Learning is just not possible”. Nonetheless, his pioneering lesion experiments provided support for the influential notion that memory is underpinned by various functionally and/or structurally connected circuits that coordinate their activity to enable the retention of information. In other words, memory is made possible by joint activity within and across neural circuits and is not localized to a single X/Y/Z neural coordinate point.

An impressive body of work emerged after Lashley’s initial attempts to isolate an engram. Rather than provide an exhaustive historical overview of engram research, here we discuss the strengths, weaknesses, caveats and controversies that have ensued in the last 10 years, beginning in the late 2000s when pioneering studies on memory introduced the genetic strategies often utilized today to study defined sets of cells and circuits processing discrete mnemonic information. Readers are referred elsewhere for thorough overviews on the historical trajectory and experimental breakthroughs that greatly influenced the modern search for the engram (Mayford, 2014; Gore et al., 2015b; Josselyn et al., 2015; Tonegawa et al., 2015; Eichenbaum, 2016).

## The State of Immediate Early Gene (IEG)-Based Mouse Models: 2006 and Beyond

In the last decade, numerous laboratories have created mouse models to genetically tag and manipulate sets of cells that were active during specific periods of time. Here, we will summarize the current models, compare the strengths and weaknesses of these models, and propose future models. While there are a number of viral strategies available that are frequently used (e.g., Gore et al., 2015a) or have been recently developed and would be promising for whole-brain engram tagging (e.g., Deverman et al., 2015; Treweek et al., 2015), we will only focus on genetically engineered mouse lines that could allow for whole-brain engram tagging, visualization and manipulation.

One of the first systems available for tagging neuronal populations was an Arc-GFP knock-in mouse line (Wang et al., 2006) in which the activity-dependent nature of the immediate early gene (IEG) Arc was leveraged to drive a fluorescent reporter. This line was influential in that it allowed for *in vivo* two-photon imaging of Arc^+^ populations; however, it did not allow for a permanent tag of a previously active neuronal population as the GFP tag was transient. Nonetheless, these mice allowed for a number of studies in the visual (Gao et al., 2010; McCurry et al., 2010) and frontal cortices (Ren et al., 2014), but imaging of deeper brain regions was technically daunting at the time. Similar lines have since been created such as the Arc:dVenus line—a line that expresses a destabilized version of the yellow fluorescent protein (YFP) under the control of the 7.1 kb of the mouse Arc promoter (Eguchi and Yamaguchi, 2009).

Next, between 2007 and 2009, a series of articles introduced strategies for tagging and manipulating cells that were active during learning and retrieval in the rodent brain (Han et al., 2007, 2009; Reijmers et al., 2007). Reijmers et al. (2007) developed an activity-dependent and inducible strategy in which cells that are active during learning become “tagged”, that is, labeled with a reporter, for subsequent visualization at a separate time point, such as during memory retrieval. This TetTag mouse is a bi-transgenic mutant that has tetracycline inducible expression of β-galactoside in activated neurons expressing the activity dependent and IEG c-Fos. The authors demonstrated that in the amygdala, cells that are active during fear learning are reactivated at levels above statistical chance during memory retrieval. Importantly, such overlap of activity is not observed in animals in which no shock was administered during the initial learning period. These findings introduced the often-used TetTag mouse line and demonstrated that overlapping amygdala ensembles are engaged during learning and retrieval of aversive events, which, by extension, argues that the amygdala processes conjunctive components of memory traces (Richards and Frankland, 2013). This line provides a long-lasting tag of neuronal activity, although it still did not achieve the permanent labeling that is necessary for assessing long-lasting memory formation and retrieval (e.g., >30-day interval).

While the former study measured endogenous activity-dependent processes, Han et al. (2007, 2009) artificially biased ~15% of amygdala neurons to become preferentially activated during fear learning and subsequently tested their contributions to behavior (see also Zhou et al., 2009). The authors first showed that increasing the transcription factor CREB in a small population of amygdala cells increases their excitability levels, which thereby is sufficient to bias these cells to become active during memory formation and retrieval compared to neighboring quiescent cells. Moreover, ablating this set of CREB-expressing cells that were biased to process a fear memory subsequently abolished the fear memory itself. Importantly, the authors showed that ablating a random amygdala ensemble of equal size did not affect memory expression. Collectively, these studies provided the tools and concepts necessary to probe the various stages of memory by modulating spatially and temporally defined neural populations across a variety of brain regions.

Following the pioneering TetTag labeling and CREB allocation systems, two laboratories created indelible labeling of memory trace using the CreER^T2^ system (Guenthner et al., 2013; Denny et al., 2014). The Luo lab created two CreER^T2^ systems using a knock-in strategy—ArcCreER^T2^ (ArcTRAP) and FosCreER^T2^ (FosTRAP). While the FosTRAP system produced clean, representative c-Fos tagging, the ArcTRAP system had a significant amount of background expression, most likely due to the knock-in strategy used. Denny et al. (2014) generated a bacterial artificial chromosome (BAC) ArcCreER^T2^ Mouse, which had low levels of background expression and indelible labeling of active cells. The utility of these mice, as opposed to the TetTag system, is that they allow for permanent tagging that is necessary for studies focusing on long-term memory, aging and disease-related states.

While we provide a representative list of IEG driver lines above, we note that each of these lines also differs by the reporter utilized. Therefore, it is crucial to characterize each reporter line in combination with the appropriate driver IEG line to obtain the cleanest representative tag of neuronal activity. For example, we have measured significant differences if, instead of a R26-LSL-eYFP line (Srinivas et al., 2001), a R26-LSL-H2B-mCherry line (Peron et al., 2015) is bred with the ArcCreER^T2^ mice (Pavlova and Denny, unpublished data). For instance, there is significantly more background expression with the H2B-mCherry line than with the eYFP line. Moreover, the concentration and timing of a tamoxifen (TAM)/4-hydroxytamoxifen (4-OHT) injection relative to the behavioral experience also produces differences in the labeled populations (Cazzulino et al., 2016). We note these important technical differences because of the following: if an active neuronal population is tagged in a manner that does not recapitulate the endogenous number of active cells in a defined period of time, then the number of tagged cells corresponding to a given experience may produce a diluted percentage. In other words, if the tagged neuronal population is significantly less than the number of neurons that is typically active following a behavioral task, then there will also be an underestimate of the ensemble size comprising a discrete memory. To address this challenge, we propose that each experimenter compare the number of active cells via protein (e.g., Arc^+^ protein) with the tagged population (e.g., eYFP^+^) to validate that the given tag represents a reliable neuronal pool.

## Future Models and Technologies

One long-sought goal for future tagging systems is the labeling and/or manipulation of multiple memory traces. A tremendous amount of progress was recently achieved by combining intersectional strategies with current IEG-based models to allow two memories of opposite valence to be visualized and artificially linked in the amygdala (Yokose et al., 2017). Building on this strategy, we propose that ArcCreER^T2^ × eYFP bigenic mice can now be combined with a viral c-Fos TetTag strategy to label four different neuronal populations. This strategy may greatly increase our current understanding of how multiple engrams are stored and interact with one another across the brain and over time, given the dynamic nature of memory processes. Another possibility is to combine an indelibly tagged population with Ca^2+^ imaging so that neuronal populations can be imaged multiple times over the course of different memory stages, such as reconsolidation and extinction (e.g., see Danielson et al., 2016). We could also begin to ask: do the tagged neurons have increased Ca^2+^ transients during memory retrieval in the dentate gyrus (DG)? Do diseases differentially affect Ca^2+^ expression in the neuronal population that was originally active during encoding? How do Ca^2+^ transients differ between tagged encoding cells vs. non-tagged cells during memory retrieval?

It would be extremely informative to create a strategy in which only neuronal populations that were active during both memory encoding *and* retrieval were optogenetically tagged (e.g., Hirsch et al., 2013; Yokose et al., 2017; provide representative intersectional strategies). As of now, most studies have only activated or inhibited populations of neurons that were active during memory encoding *or* during memory retrieval. The development of intersectional optogenetic strategies will be vital in propelling the engram field forward in the search for stable, and disjunctive, properties that define how experiences leave lasting physiological and biochemical imprints on the brain. Finally, current technologies have either allowed for optogenetic manipulation or visualization of Ca^2+^ activity occurring in neuronal populations. However, recent technologies have integrated Ca^2+^ imaging and optogenetics into the same implant (e.g., nVoke). These types of technologies will give unprecedented access to engrams—allowing for simultaneous activation, inhibition and recording of neuronal activity.

## Whole-Brain Imaging Techniques

Memories are thought to be distributed throughout the brain. Recent microscopy and immunolabeling techniques have allowed researchers unprecedented molecular access to an intact brain, which brings us closer to visualizing the neuronal architecture of an engram in its entirety. Contemporary technologies permit researchers to tag cells that are active during defined periods of time across the whole brain, to clear the tissue, and to perform immunohistochemistry without compromising much of the tissue’s structural integrity. With techniques such as CLARITY (Chung et al., 2013), PACT (Treweek et al., 2015), iDISCO (Renier et al., 2014), CUBIC (Susaki et al., 2015) and SWITCH (Murray et al., 2015), we can begin to dissect at the single-cell level the molecular and anatomical components comprising a memory, and how these properties change during the many dynamic phases of memory. These technologies, in combination with the aforementioned intersectional genetic strategies, will vastly increase our knowledge of the physical instantiation of memory storage, retrieval and updating.

## Necessity and Sufficiency Are Not Necessarily Sufficient

In recent years, numerous groups have developed novel strategies for visualizing and manipulating defined sets of cells that were either naturally or artificially biased to be active during memory formation or retrieval (Koya et al., 2009; Zhou et al., 2009; Liu et al., 2012; Guenthner et al., 2013; Ramirez et al., 2013, 2015; Cowansage et al., 2014; Denny et al., 2014; Redondo et al., 2014; Tanaka et al., 2014; Yiu et al., 2014; Gore et al., 2015a; Kim et al., 2015; Ryan et al., 2015; Cai et al., 2016; Okuyama et al., 2016; Park et al., 2016; Roy et al., 2016; Stefanelli et al., 2016; Trouche et al., 2016; Ye et al., 2016). After carefully considering the results of these studies, it was recently proposed that at least three conditions must be met for a set of cells to qualify as harboring, or at the very least processing, a component of an engram (Mayford, 2014; Josselyn et al., 2015; Tonegawa et al., 2015). First, the cells must be active at the time of encoding; second, these cells must be necessary for the neuronal and behavioral expression of memory; and thirdly, reactivating these cells must be sufficient to induce the neuronal and behavioral expression of the memory.

While these notions certainly are influential and useful for guiding theory-driven experiments that seek to find the cellular basis of engrams, the definitions of, and criteria surrounding, necessity and sufficiency are often moving targets and are contingent upon the techniques utilized to assay each. We will start with the DG to underscore each point, though we believe that the general strategy proposed translates to virtually every brain area. For instance, *in vivo* electrophysiology and histological approaches have revealed sparse activity in DG cells that are exquisitely sensitive to contextual changes (Leutgeb et al., 2007; Satvat et al., 2011). In terms of necessity, lesioning or pharmacologically inactivating the DG often impacts memory encoding but does not have an impact on retrieval (Lee and Kesner, 2004); optogenetically silencing the DG has a similar effect and impairs memory encoding but not retrieval (Kheirbek et al., 2013); yet, chemo- and optogenetically inhibiting ~6%–9% of DG cells that were previously active during learning, but not a similar fraction of randomly active DG cells, impairs memory encoding *and* retrieval, thus unmasking the real-time necessity of putative engram cells for these stages of memory (Denny et al., 2014; Park et al., 2016).

Based on these data, we believe that asking the question, “Is the DG necessary for memory encoding and/or retrieval?” perhaps can be posed in a more nuanced manner that provides a framework for reconciling seemingly conflicting data: “Under what conditions is the DG necessary for memory encoding and/or retrieval?” Such a reframing can also be applied to other brain regions that are involved in mnemonic processes, including CA1 (Tanaka et al., 2014; Okuyama et al., 2016), amygdala (LeDoux, 2000; Gore et al., 2015a; Rashid et al., 2016), retrosplenial cortex (Cowansage et al., 2014), nucleus accumbens (NAcc; Britt et al., 2012; Pascoli et al., 2014; Xiu et al., 2014), prefrontal cortex (Ye et al., 2016) and piriform cortex (Choi et al., 2011). At present, the data suggest that the length of inhibition, the number of cells inhibited and the time at which they are inhibited in relation to memory encoding and retrieval are all crucial factors to consider when interpreting such interventions’ contributions to circuit activity and behavior.

A similar line of reasoning exists for sufficiency experiments as well. For instance, optogenetically activating the majority of the DG impairs memory encoding and retrieval (Kheirbek et al., 2013; Stefanelli et al., 2016); yet, optically activating ~4%–6% of DG cells that were previously active during memory formation induces the behavioral expression of the associated memory (Liu et al., 2012; Stefanelli et al., 2016). These discrepancies are not conflicting; rather, they highlight the conditions under which the activity of the entire DG can impair memory while a subset of DG cells are sufficient to facilitate encoding and/or activate retrieval. Thus, the diverging experimental parameters utilized even within the same subregion profoundly influence the ensuing phenotypes. Similar to the aforementioned necessity experiments, notable differences across sufficiency studies include the number of cells activated, the prior history of each cell population manipulated, and the timescale of each perturbation.

Correspondingly, long-term and short-term inhibition and activation of neural circuits produce markedly different systems- and behavioral-level states, and can also produce acute off-target effects that confound interpretation of a given brain region’s involvement in producing a given phenotype. The brain states resulting from acute and chronic perturbations requires careful consideration, given that either timescale may differentially recruit compensatory mechanisms, such as bringing downstream brain areas “online” both in real-time and over time, which themselves are capable of supporting a given behavior (Goshen et al., 2011; Otchy et al., 2015). However, these caveats can be leveraged experimentally, and often elegantly. For instance, Trouche et al. (2016) utilized an activity-dependent and inducible genetic strategy to tag 6% of CA1 pyramidal neurons that were previously active in a cocaine-paired environment. Optogenetically inhibiting these CA1 cells triggered a separate subset of once-quiescent CA1 neurons to become active. The newly surfaced real-time activity of the previously dormant CA1 cells enabled an alternative hippocampus map to emerge, which was then used to recode a cocaine-paired environment with the neutral alternative map. Strikingly, the animals subsequently showed a complete abolishment of cocaine place preference. These promising findings take advantage of off-target effects resulting from rapid perturbations and provide credence for the notion that artificially modulated mnemonic processes—and their acute and sometimes unexpected impact on circuit-level processing—can be utilized to alleviate maladaptive states, a topic which we discuss below.

## Necessity and Sufficiency Are Not Always Opposites

When planning experimental schedules, it is noteworthy that necessity and sufficiency are not always diametrically opposed. In the DG, if one were to base an experimental strategy solely on previous literature, then its role in being sufficient for memory retrieval may have gone overlooked because a number of studies have argued that it is not necessary in memory retrieval (Kheirbek et al., 2013). We argue that when a behavior is said to be independent of a brain area, it does not follow *a priori* that the brain area is not important for that behavior under all conditions; it follows that the brain area is dispensable given a defined set of conditions. The same holds true for sufficiency: a brain area may fail to be sufficient to activate a behavior while still being necessary for the behavioral phenotype. Finally, and for future experiments, tasks traditionally thought to be independent of a brain area may indeed not require its functional integrity, but if an experiment revealed that a given brain area was sufficient to modulate a specific behavior despite being dispensable for the task, we believe that its importance would not be diminished, but nuanced.

## The Many Ways to Start a Car

A common criticism of engram research is that the cellular perturbations utilized are highly artificial and do not mimic endogenous activity, such as recapitulating precise temporal sequences known to exist in areas such as the hippocampus. Therefore, the ensuing phenotype that we call memory retrieval may or may not be mnemonic in nature and may simply be a non-specific behavioral output. To address this issue, we propose a simple conceptual bifurcation for probing questions about engrams and what a given set of experiments aims to investigate:

(1) How *does* memory work?
(2) How *can* memory work?

To answer question 1, observational tools such as *in vitro* and *in vivo* electrophysiology, fiber photometry, voltammetry, calcium imaging and unbiased histological assessments are well suited to reveal the endogenous mechanisms supporting memory (Gunaydin et al., 2014). To answer question 2, interventional tools such as optogenetics, chemogenetics and pharmacology are well equipped to modulate endogenous processes and to test just how far a given system can be pushed to still yield a meaningful phenotype. The findings of both questions can feedback on one another to guide future experimental strategies.

This framework can be applied to areas outside the hippocampus and forms the basis for much research seeking to rewire the brain. For instance: how do visual and auditory cortices work, and how can they work? A rich body of research on visual and auditory cortex has revealed the existence of retinotopic and tonotopic maps with an extraordinary capacity for processing both simple and complex stimuli (for a review see Leamey and Sur, 2001; Sanes and Zipursky, 2010). Building on how these areas normally function, a landmark study rewired retinal projections to auditory cortex and showed that these redirected inputs enabled functional and behavioral responses to visual stimuli (von Melchner et al., 2000), thus highlighting how these two areas *can* work given that such cross-modal projections have to be artificially induced.

Or, for instance, how does the VTA dopaminergic system work and how can it work? Guided by the former, many groups have recorded changes in phasic activity of genetically identified VTA dopaminergic neurons in response to various task demands or prolonged periods of stress (reviewed in Tsai et al., 2009; Cohen et al., 2012; Lammel et al., 2013; Steinberg et al., 2013). Guided by the observed physiology, recent optogenetic strategies have attempted to mimic or reset the endogenous temporal activity of the VTA to alleviate maladaptive states. For example, phasic optogenetic activation of VTA dopaminergic neurons results in robust changes in depression-related behavior after stress protocols of varying severity and durations (Chaudhury et al., 2013; Tye et al., 2013; Friedman et al., 2014). We speculate that the length of each stimulation protocol used perhaps pushes multiple downstream circuits into unnatural states that may or may not reflect a brain state that would result from natural VTA functionality. Still, while the VTA may not inherently fire five spikes every 10 s at 20 Hz for 10 min (Chaudhury et al., 2013), or eight pulses at 30 Hz every 5 s for 3–15 min (Tye et al., 2013), the fact that these perturbations nonetheless have profound impacts on neuronal activity and behavior underscores the importance of revealing how the VTA can work both in terms of circuit-level activity and behavioral readouts. In our opinion, these sophisticated studies highlight the brain’s extraordinary flexibility to respond to a wide array of exogenous influences. One only needs to apply the same logic to virtually every pharmacological vehicle utilized to treat pathologies of the brain to appreciate that unnatural interventions can still yield insightful outputs. Pharmacological agonists do not precisely and fully mimic endogenous activity in the brain either, and yet they can have profound influences in resetting circuits away from a maladaptive state.

A similar line of reasoning applies to hippocampus and amygdala engram studies. A common criticism of Liu et al. (2012) and a series of related articles modulating the DG is that artificially activating tens of thousands of DG cells simultaneously at 20 Hz with 15 ms pulse widths for 3 min is not how the DG naturally fires during memory retrieval. This is almost certainly true. We argue, however, that these experiments provide evidence for how the hippocampus *can* work and not necessarily for how it *does* work. While minutes-long stimulation of a fraction of cells at 5 Hz in the retrosplenial cortex (Cowansage et al., 2014), 20 Hz in the DG (Liu et al., 2012; Ramirez et al., 2013; Redondo et al., 2014; Ohkawa et al., 2015; Ryan et al., 2015; Roy et al., 2016; Stefanelli et al., 2016) and 20 Hz in the basolateral amygdala or ventral CA1 (Yiu et al., 2014; Gore et al., 2015a; Okuyama et al., 2016; Rashid et al., 2016) does not recapitulate endogenous physiological firing patterns, we believe that these findings yield powerful strategies for artificially commandeering mnemonic processes and for navigating memory’s largely unexplored capacity to be externally controlled (for reasons discussed below). Notably, recent work has demonstrated that the DG increases in beta amplitude (15–30 Hz) in an associative learning task, perhaps reflecting an oscillatory shift in processing states that 20 Hz DG stimulations partly capture and/or recapitulate (Rangel et al., 2015). This conjecture remains to be tested by recording physiological activity both within and downstream the DG during optogenetic stimulation.

The same logic applies to molecular interventions. For instance, overexpression of the transcription factor CREB can be used to increase neuronal excitability, which permits memories to be allocated, erased, or reactivated both opto- and chemogenetically via a defined set of basolateral amygdala or hippocampus cells (Han et al., 2007, 2009; Zhou et al., 2009; Yiu et al., 2014; Cai et al., 2016; Park et al., 2016; Rashid et al., 2016). A common criticism of these studies, however, is that overexpression of CREB is not natural and there has yet to be a demonstration that these mechanisms exist intrinsically to allocate memories. Such an argument is valid if one aims to use these studies as examples of how memories naturally work, because we do not know yet if this is, in fact, how memories work. But, the criticism is moot when one considers these studies as examples of how memory can work, because the CREB strategies utilized have nonetheless been effective at allocating and manipulating various components of engrams, including fear and reward responses. Thus, the insight gained by unnaturally perturbing memory is not undermined but underscored.

Still, a vast array of literature suggests that the temporal structure of an experience is reflected in the activity of neuronal ensembles, including in the hippocampus (MacDonald et al., 2011). As such, artificially recapitulating natural firing patterns may produce three outcomes: it is not effective, it is just as effective, or it is more effective at activating various components of a memory (Häusser, 2014). The latter conjecture is supported by recent data in CA1, in which neuronal ensembles display precisely timed sequences of activity in response to spatial-temporal relational processing, which together are believed to comprise the global structure of an experience (Wood et al., 1999; MacDonald et al., 2011; Buzsáki and Moser, 2013; Eichenbaum, 2016). Despite the success of a 20 Hz stimulation protocol in the DG, Ramirez et al. (2013) failed to observe light-induced memory retrieval in response to the same 20 Hz protocol in CA1. However, the authors did not attempt to recapitulate more endogenous firing patterns, as have been previously observed during fear memory retrieval. Seidenbecher et al. (2003) reported that fear memory retrieval synchronizes CA1 and the lateral amygdala at the type II theta (4–8 Hz) band, thus successfully identifying a neural correlate of conditioned fear. Guided by these experiments, Ryan et al. (2015) optogenetically utilized a 4 Hz stimulation protocol to reactivate CA1 cells previously active during fear memory formation, which led to robust fear memory retrieval, despite not recapitulating the exact sequence of activity within a CA1 ensemble. These data also complement recent findings that 4 Hz synchrony between areas such as the amygdala and prefrontal cortex control memory expression and that 4 Hz stimulation of prefrontal cortex is sufficient to drive freezing behavior (Dejean et al., 2016; Karalis et al., 2016). Collectively, these studies highlight the importance of utilizing physiological data from research aimed at discovering “how does memory work” to guide interventional experiments aimed at answering “how can memory work” and vice versa.

Moreover, the percentage of cells tagged by an experience and subsequent stimulation parameters can jointly influence whether or not optical stimulations induce memory expression. For example, Ohkawa et al. (2015) successfully utilized a 20 Hz stimulation protocol in CA1 to link a context-specific, hippocampus-mediated memory with unconditioned stimulus-responsive cells in the basolateral amygdala, thus leading to the formation of an artificial associative memory. It is noteworthy that Ohkawa et al. (2015) tagged ~13% of CA1 cells with a lentivirus ChR2 vector, whereas Ramirez et al. (2013) tagged ~50% of CA1 cells with an AAV9 ChR2 vector; we speculate that the spatial specificity of labeling a smaller proportion of cells with the former strategy managed to capture more “signal” in terms of CA1 context specific cells whereas tagging ~50% of cells in the latter case perhaps captured more “noise” during the labeling period and thus failed to unmask a behavioral response. Therefore, a boundary condition for achieving context-specific reactivation in CA1 appears to be both frequency of stimulation and number of cells tagged.

In summary, observational techniques provide excellent strategies for understanding how does memory work. Interventional techniques provide excellent strategies for answering how memory can work when the perturbation parameters go outside the realm of endogenous firing patterns. We believe both approaches greatly advance our understanding of the brain equally (e.g., Nabavi et al., 2014). With regards to engram research, we propose that the capacity to artificially induce memory retrieval is as encouraging as it is effective, but it does not follow that this is how memory works. By analogy, sufficiency studies often hotwire the car and bypass the natural ignition; extraordinarily, the car starts nonetheless.

## But Is It Really a Memory?

Contemporary intersectional strategies, projection- and target-specific perturbations, and large-scale imaging techniques provide powerful methods for interrogating the cellular landscape supporting memory (Lerner et al., 2016; Rajasethupathy et al., 2016). When this landscape is activated in rodents, memory retrieval can manifest itself through an impressive repertoire of behavioral readouts. Here, we will focus on freezing behavior because of its profound influence in memory research and the abundance of studies devoted to delineating its underlying circuitry.

There are many ways to induce freezing in a rodent. Each route presumably is underpinned by specific circuit activity that may or may not contain mnemonic information. For example, it is possible to induce freezing by activating a variety of brain areas and projections, including the hippocampus (Liu et al., 2012), lateral, basal and central amygdala (Ciocchi et al., 2010; Johansen et al., 2010; Gore et al., 2015a), periaqueductal gray (Tovote et al., 2016), motor and primary sensory cortices (Kass et al., 2013), prefrontal projections (Rajasethupathy et al., 2015) and retrosplenial cortex (Cowansage et al., 2014). Importantly, if mnemonic information is conjured up when reactivating a brain area, each of these findings makes specific hypotheses with regards to the phenotypic consequences resulting from acute and chronic modulation of each area (Feldman Barrett and Wager, 2006; LeDoux, 2012). We turn to four examples—the hippocampus, amygdala, prefrontal cortex and sensory cortex—to highlight the triumphs and challenges that come with using freezing as a measure of memory retrieval.

If mnemonic information is artificially activated, then it becomes possible to design experiments that directly measure the qualitative nature of the information that has come “online”. In the hippocampus, optogenetic stimulation of cells that were previously active during fear learning is sufficient to reactivate downstream neuronal ensembles that were also originally active during fear learning, arguing that these neural ensembles have structurally and/or functionally wired together—a cellular correlate of a conjunctive memory trace (Hebb, 1949; Ramirez et al., 2015; Ryan et al., 2015). Likewise, optogenetic inactivation of a defined set of neurons that were previously active during memory formation both disrupted retrieval and prevented the reinstatement of cortical representations of the memory (Tanaka et al., 2014). Such stimulations are also sufficient to reactivate the behavioral expression of fear, even in the presence of multiple memories but with only one fear memory “tagged” (Liu et al., 2012). Within the same region, activating cells that were previously active during neutral memory formation is sufficient to act as a context-specific conditioned stimulus (Ramirez et al., 2013) and activating cells previously active during positive memory formation is sufficient to induce reward-related behaviors (Redondo et al., 2014). These hippocampus cells, importantly, undergo plasticity related changes including increases in dendritic spine density, excitability and AMPA/NMDA ratios when compared to neighboring quiescent cells (Ryan et al., 2015). Based on these data, we hypothesize that the mnemonic information that these stimulated circuits are bringing “online” is contextual, at the very least, in nature.

If true, these data make the prediction that chronic stimulation of these hippocampus cells may elicit context-specific modulation of the associated memory. In one experiment, animals were fear conditioned in environment A and also in environment B, but only cells active during the former were tagged in the hippocampus with ChR2 (Chen and Ramirez, unpublished data). Next, these cells were optically multiple times across five days. Animals showed low freezing levels when placed back in environment A while freezing levels remained high and comparable to control levels in environment B, arguing that chronically stimulating these cells elicited a context-specific extinction-like phenotype. These data resonate with findings from Cowansage et al. (2014): by tagging cells active during neutral memory formation in context A, followed by re-exposing the animals to context A one day later and fear conditioning them, or exposing them to novel context B, the former group showed light-induced fear behavior while the later did not. Strikingly, reactivating these retrosplenial cells drove fear behavior in a context-specific manner independent of the hippocampus and in a manner that was also sufficient to recapitulate activity in downstream areas originally active during memory formation, such as the amygdala and entorhinal cortex. These influential data support the proposed existence of context-specific memories stored redundantly in cortical-hippocampal areas (McKenzie and Eichenbaum, 2011; Tayler and Wiltgen, 2013).

Would similar results occur if the aforementioned extinction experiments were performed in other brain areas (Mayford, 2014)? If we consider the amygdala, the many ways to induce freezing become strikingly clear: for instance, Ciocchi et al. (2010) and Johansen et al. (2010) observed light-induced unconditioned freezing responses when stimulating the lateral amygdala and central medial amygdala, respectively; Gore et al. (2015a) elicited freezing responses by optically activating cells in the basolateral amygdala that were previously active during fear conditioning; and, Tovote et al. (2016) characterized a freezing pathway from the central amygdala to the ventrolateral periaqueductal gray to motor areas, all of which are sufficient to modulate freezing responses. Now, if the aforementioned extinction experiments in the hippocampus were instead performed in these amygdala subregions (i.e., chronically stimulating amygdala cells across multiple days), we hypothesize that chronic stimulation would elicit an overall reduction of fear behavior; in other words, the neuronal wiring of these areas perhaps is biased to process specific valences and would thus demonstrate valence-specificity (i.e., reduction of fear in environment A *and* B) as opposed to context-specificity (i.e., reduction of fear in environment A *or* B). Indeed, neuronal ensembles that display valence specificity have been shown to exist in both the amygdala and NAcc, where cells active during negative experiences are preferentially reactivated during subsequent negative experiences, and the same is true for positive experiences (Redondo et al., 2014; Xiu et al., 2014; Gore et al., 2015a; Namburi et al., 2015; Beyeler et al., 2016; Kim et al., 2016). Such preferential wiring indicates that associative learning is processed through innate circuits capable of linking negative or positive emotions to neutral sensory stimuli in the service of biasing behaviors in an adaptive manner (Janak and Tye, 2015).

In the same way that the amygdala and NAcc show preferential topographies for processing valence, and given the highly processed multi-modal information traveling in cortico-hippocampus circuits, we speculate that these circuits exhibit preferential wiring for keeping a global record of spatial-temporal relationships in the service of guiding future memory-based decisions (Wood et al., 1999; Buzsáki and Moser, 2013; Place et al., 2016). In support of this hypothesis, in DG, CA3 and CA1, exposure to the same context twice elicits an above-chance level of overlap in activity whereas exposure to two different contexts elicits either below chance-level overlap, chance-level overlap, or above chance-level overlap but at a magnitude lower than exposing an animal to the same context twice (Guzowski et al., 1999; Guzowski, 2002; Satvat et al., 2011; Liu et al., 2012; Deng et al., 2013; Ramirez et al., 2013; Tayler et al., 2013; Denny et al., 2014; Cazzulino et al., 2016). Testing whether or not newly developed tagging strategies can unveil context-specificity in areas downstream of the hippocampus or valence-specificity within cortico-hippocampus circuits remains an exciting area of inquiry, both within and across brain regions (Adhikari et al., 2010; Fanselow and Dong, 2010; Kheirbek et al., 2013; Wang et al., 2013; Ciocchi et al., 2015). We propose that all of these nodes process critical components of a distributed engram.

A similar line of reasoning can be applied to a newly discovered top-down projection from anterior cingulate cortex to the hippocampus (Rajasethupathy et al., 2015; for a newly discovered dorsal hippocampus to prefrontal cortex projection, see Ye et al., 2016). Rajasethupathy et al. (2015) find that stimulating this projection after, but not before, fear conditioning elicits freezing behavior. This remarkable finding demonstrates that the capacity to induce freezing at this projection is experience-dependent but it is yet to be determined whether or not manipulating this projection would elicit context specific behavioral outputs—the latter can be tested through similar extinction-related experiments as discussed above. Another possibility is that the light-induced freezing observed is a result of manipulating a circuit that is directly processing pain/shock-related sensory information, and the same alternative explanation applies to the light-induced freezing observed in Liu et al. (2012). To test whether or not shock-related information is directly contributing to light-induced freezing, one can utilize an immediate shock protocol, in which an animal generally does not have enough time to associate a context with a shock, to note if light-induced freezing still occurs after such behavioral schedules. In the hippocampus, multiple follow-up experiments have ruled out this possibility (Ramirez et al., 2013; Ryan et al., 2015), and future experiments in the ACC-CA1 projection are ripe for similar analyses.

Context specificity might also be achieved by combining projection-specific manipulations with activity-dependent tagging strategies. Promisingly, within the prefrontal cortex, using c-Fos-driven tagging strategies, Warren et al. (2016) show that ventromedial prefrontal cortex mediates both operant reward and extinction memories intermingled within the same cortical area—a finding that previously eluded researchers attempting to manipulate the prefrontal cortex globally as opposed to stimulating previously active ensembles. Such intersectional strategies have been recently leveraged to resolve the wiring and molecular properties of cells associated with distinct experiences in the prefrontal cortex: one recent study found that prefrontal cell populations differ in their causal impact on behavior, long-range wiring and gene expression profiles (Ye et al., 2016). Interestingly, cells expressing the IEG NPAS4 in the prefrontal cortex were preferentially recruited for positive experiences, suggesting the existence of either hardwired prefrontal circuits that capture positive experiences or that these cells relay positive information to and from areas such as the basolateral amygdala and NAcc, which together may be critical and innate loci for processing discrete valences.

Finally, if we turn to the sensory areas, such as the retina, it has been speculated that activating retinal cells previously active during fear learning would also be sufficient to elicit freezing (Mayford, 2014). However, if light-induced freezing is observed, then one can test the nature of the mnemonic information brought online (if any) experimentally: one can pair a visual stimulus (green) with a shock such that the animal learns that green predicts shock; next, one can chronically activate the “green” responsive retinal cells in an attempt to extinguish the fear memory. We make three predictions: either this does nothing, this modulates the fear responses to green but leaves the contextual memory intact, or activating “green” leads to the activation of the contextual memory and fear responses to both are modulated. One can also conceive of experiments in which stimulating “green” (or any other sensory modality in an activity-dependent manner) and recording from hippocampus engram cells to note if natural and/or artificial sensory activation leads to the reinstatement of a hippocampal trace as well.

It is important to note, however, that recent data supports the notion that activating freezing responses via hippocampal and cortical networks is experience dependent and not a hardwired response. In our speculative sensory cortex experiment, if we inactivate the representation of “green” prior to training, the animal presumably would not be able to perceive green anymore and thus green-shock pairings would fail to produce an associative memory. However, in the hippocampus, blocking the activity of one contextual representation does not prevent learning from occurring; moreover, tagging DG cells active during fear learning but simultaneously blocking upstream CA1 activity prevents subsequent light-induced freezing from occurring when DG cells stimulated. These lines of data argue that these circuits are not hardwired to produce a freezing response (Tanaka et al., 2014; Ryan et al., 2015; Stefanelli et al., 2016; Trouche et al., 2016). Of the numerous brain areas involved in processing memories, the crucial sites of plasticity for contextual representations so far include c-Fos-expressing DG cells and crucial sites of plasticity for context-shock associations include lateral and basolateral amgydala cells. We conclude that both are crucial nodes of an otherwise distributed engram.

## The Brain’s Inconvenient Truth

Our terminology has meandered between “brain regions” and “brain areas” rather liberally. However, no single brain region evolved in a vacuum and recent projection-specific analyses have demonstrated the remarkable heterogeneity that exists both within structures and the differential role their axonal outputs often play. For instance, the hippocampus contains projection neurons that synapse onto the amygdala, prefrontal cortex and NAcc, and each projection fires preferentially to fear, anxiety and reward-related behaviors, respectively (Bagot et al., 2015; Ciocchi et al., 2015). Single hippocampal output neurons have also been shown to bi- and trifurcate to synapse onto these same areas. Moreover, the amygdala contains genetically distinct and spatially segregated populations that project to the central amygdala and NAcc that both code for aversive and reward-related states (Kim et al., 2016). Among its many outputs, the prefrontal cortex sends projections to the lateral habenula and raphe nucleus to differentially facilitate effortful behavioral responses (Warden et al., 2012). Thus, circuit elements comprise mono and polysynaptic webs of dynamic activity recruited differentially for various task demands. The hints of such preferential wiring are emerging rapidly, such as lateral habenula axons synapsing onto prefrontal cortex-projecting VTA cells or ventral HPC cells differentially targeting basolateral or central amygdala cells with distinct functional properties (Lammel et al., 2013; Xu et al., 2016).

Thus, when relating structure to function, structure here perhaps is best viewed as a shifting wiring diagram with genetically biased topographies existing based on receptor topology, projections and distinct types of informational processing capacity. Such landscapes are currently being delineated in the hippocampus—for instance, recent evidence suggests that cells that are active during learning in entorhinal cortex, DG and CA3, are all preferentially connected within hours to each another and not to neighboring quiescent cells (Ryan et al., 2015; Roy et al., 2016). These data open up the exciting possibility of extant genetically defined and projection-specific routes of informational flow for spatial, temporal and emotional processing of memories. For instance, could positive or negative engram cells in the hippocampus preferentially project to amygdala cells that themselves preferentially project to NAcc D1- or D2-expressing cells, respectively? We speculate that such landscapes are the rule, not the exception, but there undoubtedly exists a spectrum of plasticity in these circuits to confer the flexibility that learning demands (Redondo et al., 2014). Together, these studies highlight the tremendous complexity present in neural circuits; the prospect of visualizing and manipulating independent features comprising a memory is promisingly underway.

## A Cross-Species Approach to Engram Research

We begin our discussion of cross-species approaches to engram research on a brief note. The quest to understand the encoding, retrieval and modification of engrams is deeply translational by nature. Across the evolutionary ladder, do organisms make, use, and modify memories with shared mechanisms? By studying cellular representations of episodic memory traces in a rodent, are findings to these questions applicable to the human brain and various patient populations?

“Translational” is both a buzzword and a hot button in science: its frequent use can often be an application of lip service for funding agencies, for instance. Scientific labs are inherently specialized entities and true translational research requires work across the entire molecular, cellular and behavioral continuum of both non-human and human studies. Very few labs exist with the expertise and the resources to cover this expansive ground alone. To begin to make translational research more tractable, labs may pose questions outside of what might be their immediate scientific comfort zone, including utilizing multi-disciplinary approaches and performing cross-species analyses. Productive collaborations must be forged and funded, and labs must be proactively engaged with cross-species colleagues to determine that their experimental designs are most relevant and most impactful. The resulting translation can occur in two directions: backward from the human or forward from the cell or animal. In this section, we argue that both need to occur and that forward translation is maximally impactful when anchored around a conceptual framework driven by human neuroscience.

## A Conceptual Framework for Cross-Species Engram Research

Memory research holds enormous promise for furthering our ability to understand how the healthy brain allows us to form and use memories, and to delineate the mechanisms underlying memory impairments in states of aging, disease and psychiatric disorders (Tonegawa et al., 2015). We propose that translation can begin with a general top-down approach that starts with human cognitive neuroscience data to motivate more targeted work in *in vitro* systems and animal models to permit construct and face validity. From this data, a list of candidate brain regions and pathways for further exploration can be generated. Nonhuman primates can then serve as a crucial back-translational link to pair human tasks, methodology, and endpoints with cellular and systems levels approaches (e.g., *in vivo* electrophysiology, stimulation, lesion) to obtain detailed mechanistic information (e.g., spikes and coherence) regarding circuit activity in brain regions of interest derived from human studies. Finally, the rodent represents the convergence of extremely powerful and selective genetic labeling techniques with the similar high-resolution cellular and systems level approaches that can be employed in the primate. Therefore, guided by human work, primate research can focus inquiry towards particularly relevant brain circuits in health and disorders, while rodent work can inform this translational dialog with the exquisite cell-type observations and interventional toolkits available.

The clear strength of work in humans is that they are humans, and they therefore serve as the yardstick that all work in non-human species and cells may attempt to approximate. In particular, the repertoire of human behavior and cognition is rich and can manifest manifold, such as through self-reports and brain scans (Buckner and Krienen, 2013). With regards to the latter, one major strength of human work is that task-related fMRI can be applied to obtain a real-time picture of a brain engaged in memory processes at a whole-brain resolution (Matthews et al., 2006). The limitation of fMRI is that BOLD effects are driven through neurovascular coupling and the observed changes in the BOLD signal occur at rather large timescales compared to the millisecond timescales on which the cells of the brain operate (Kahn et al., 2013). Therefore one cannot directly ascribe a change in BOLD signal to neuronal activity *per se*, but one can use other convergent evidence from human and animal literatures to intuit what a change in a BOLD signal in a given paradigm might reflect (Schölvinck et al., 2010; Kahn et al., 2013). In this regard, research focusing on the human brain can delineate the structural and functional properties of engrams at a macroscopic level, while animal work is critical for defining the microscopic landscape governing the dynamic stages of memory (Ranganath and Ritchey, 2012).

Our ability to invasively interrogate the human brain, particularly from a controlled and systematic standpoint, is limited relative to other species. However, structural and functional MRI has allowed us to define deficits specific to certain patient populations and processes (e.g., entorhinal cortical and hippocampal deficits in preclinical and clinical Alzheimer’s Disease [AD]; Small et al., 2011; Jagust, 2013; Khan et al., 2014), certain medical procedures (e.g., frontal lobotomy, cingulotomy; Ballantine et al., 1987) and electrical stimulation (Penfield and Rasmussen, 1950) have allowed us to begin ascribing function to human brain regions, and techniques such as transcranial magnetic stimulation (TMS) has allowed us a reversible window into cortical function (Optiz et al., 2016). One further weakness is that humans are not genetically tractable insofar as labeling and manipulating brain circuits in a cell-specific manner, although genetic studies of tremendous value can be done to establish baseline, intermediate, and treatment-responsive (and resistant) phenotypes (Insel and Cuthbert, 2009; Thompson et al., 2014).

Work in nonhuman primates is an essential translational conduit that will enable the most targeted and relevant back-translational questions to be posed in rodents and ensure that our findings from rodents have maximal translational impact at the human level (Jennings et al., 2016). Short of work in select human patient populations, nonhuman primates are the closest representative system of the human brain, particularly when it comes to memory research and with the recent surge in genome editing interest, strategies to generate transgenic primates are being actively pursued (Jennings et al., 2016). In this regard, nonhuman primates allow controlled studies to be done that utilize very similar or identical methodology and experimental procedures to human studies paired with manipulations (e.g., *in vivo* electrophysiology, stimulation, lesions) commonly utilized in rodent studies (Zola et al., 2000; Jutras and Buffalo, 2010; Gil-da-Costa et al., 2013). For example, noninvasive eye-tracking methodology has been utilized both in rhesus macaques as well as human amnestic Mild Cognitive Impairments (aMCI) and AD patients to demonstrate equivalent engagement of the hippocampus in visual object recognition paradigms (Crutcher et al., 2009; Jutras and Buffalo, 2010; Meister and Buffalo, 2016). In another study, Gil-da-Costa et al. (2013) utilized scalp-based EEG to demonstrate the homology of event-related potentials (ERPs) between healthy human volunteers and rhesus macaques in an auditory oddball paradigm. This homology subsequently allowed the authors to demonstrate equivalent effects of ketamine on ERPs in parallel in both rhesus macaques and humans, suggesting that this approach could serve as a viable translational model for sensory processing deficits in psychiatric disorders.

We hold that in the near future the findings of optogenetics will be leveraged as conceptual scaffolds to study the human brain for successfully treating neurologic and psychiatric disorders (Song and Knöpfel, 2016). This first application of optogenetics in humans is a treatment trial for retinitis pigmentosa that is currently recruiting (RetroSense Therapeutics, 2015). However, as clinical efficacy in humans remains to be realized, we argue here for a more immediate translational application of memory research. An area of immediate impact that engram research can have is in providing a platform for pharmacologically and non-invasively modulating the neural mechanisms underlying memory. From a therapeutic standpoint, the beneficial and detrimental ways to modulate encoding, retrieval, and updating mechanisms can then begin to be delineated when aligned with human and primate functional neuroscience data.

Although the recent development of optogenetics has allowed the labeling and causal manipulations of cells processing discrete experiences, arguably one of the most frequently applied means for encoding, reactivating and modulating memories *in vivo* has been the use of small molecule drugs. Indeed, many recent advances described below have been made in the development of selective small molecule tools that can promote or antagonize endogenous transmitter or neuromodulator activity, providing a degree of physiological stimulation in a brain-wide manner that optogenetics cannot yet achieve (Conn et al., 2009a,b, 2014; Changeux and Christopoulos, 2016). While drug studies are often conducted with the aim of improving a behavioral readout of memory, for instance, or otherwise improving nervous system functioning, we must also pay careful attention to how a drug mechanism of interest is shaping brain structure and function at a microcircuit and systems-level.

**Figure 1:**
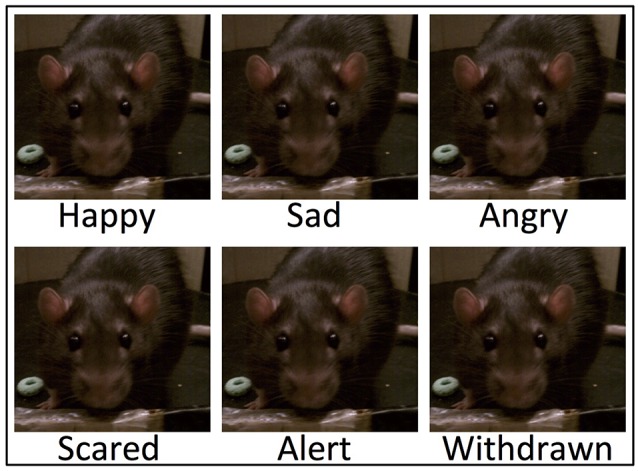
**How is my rat feeling?** Is all behavior is useless? No. But when attempting to model a human behavior in an animal it can either be absent altogether, misleading due to anthropomorphic interpretations, or paint an incomplete picture because the behavior is partially conserved evolutionarily. Therefore there is value and merit in examining brain circuits independent of, and in addition to, behavior in a complimentary fashion by leveraging the strengths of convergent approaches across multiple levels of analysis.

We propose that, given the advent of genetic models and tools discussed here, *in vivo* pharmacology and deep-brain stimulation studies in humans can and should move beyond studies that focus solely on behavior. Behavior is powerful, but it should not be seen as the end-all for a given neuronal or circuit-level manipulation. Indeed, in mental illnesses such as schizophrenia and AD, behavioral abnormalities can manifest years after maladaptive neural signatures have begun to emerge, such as cortical thinning and abnormal plaques (Insel, 2010). Along these lines, Kaiser and Feng (2015) propose a focus on genes and circuits as well as behavior to make translation possible while carefully avoiding anthropomorphizing any putative behavioral index of maladaptive states. As a thought experiment, a person can be sitting still for 1 min and yet undergo a score of positive and negative emotions, but one might not always be able to tell simply by reading out his or her behavior. Analogously, a mouse can undergo a variety of internal state changes without these changes manifesting as behaviors, perhaps because many traditional assays utilized are not always sensitive enough to tease out a casual thread between physiological activity and the particular behavioral paradigm utilized (Figure 1). In our opinion, shifting to genes and circuit-based readouts might tremendously compliment changes in behavior, but instances exist where dramatic changes to physiology do not produce an obvious behavioral change. For instance, unilateral inhibition of ventral hippocampus terminals onto the prefrontal cortex did not affect avoidance behavior, but it did disrupt phase locking of prefrontal spikes to ventral hippocampus theta to a similar degree as did bilateral inhibition (Padilla-Coreano et al., 2016). The latter, however, also produced avoidance-like behavior and thus, as the authors note, unilateral inhibition successfully separates the physiological and behavioral effects of disrupting ventral hippocampus to prefrontal cortex inputs. Thus, modern neuroscience has the capability to peer inside brain circuits and systems with single-cell and cell-type resolution to observe the rich landscape of physiological mechanisms underlying, though sometimes dissociating from, a prescribed set of complex behaviors.

## Drug Development and Memory Research

In general, drug development is still largely target-driven (Pangalos et al., 2007). Once one identifies a target (e.g., through a combination of animal knockout studies with human genetic or expression studies) the next question is how to modulate this target to obtain a desired therapeutic effect (e.g., to improve memory). If two drugs that activate the same receptor through different mechanisms of action but both improve memory in healthy animals, what are the mechanisms and network properties facilitating such a phenotype? Is it reasonable to expect that these drugs could be given to the same population of patients? Based upon behavioral pharmacology data alone, the answer would seem to be yes. However, a recent *in vivo* electrophysiology study highlights the notion that drugs may have convergent influences on behavior while having dramatically different influences on physiology and vice versa. The authors recorded CA3 and CA1 hippocampal place cell activity in rats in the presence of two different types of M_1_ muscarinic acetylcholine receptor (mAChR) activators that are known to improve measures of hippocampal memory: an M_1_ positive allosteric modulator (PAM) was compared to an M_1_ agonist (Digby et al., 2012; Galloway et al., 2014; Lebois et al., 2016). Both were also compared to the acetylcholinesterase inhibitor (AChEI) donepezil, which is standard for Alzheimer’s treatment. M_1_ PAMs and agonists both activate the M_1_ receptors; however agonists are direct activators (independent of acetylcholine), whereas PAMs potentiate the action of acetylcholine when it is bound to the receptor specifically (Conn et al., 2009a,b, 2014; Changeux and Christopoulos, 2016). In this regard, agonists elicit tonic activation, whereas PAMs are active only in the presence of acetylcholine and thereby preserve the endogenous spatial and temporal properties of M_1_ signaling.

While at first this might seem to be an esoteric distinction since they both improve memory, these two activators were found to elicit markedly different effects on hippocampal network physiology. Indeed, the M_1_ PAM and donepezil biased hippocampal processing toward pattern separation and the formation of orthogonal memory traces, whereas the M_1_ agonist biased hippocampal processing toward pattern completion and the emphasis of previously coded memory traces. Understanding such pharmacological effects on network physiology in the awake behaving brain will be critical for giving us the ability to rationally target therapeutic mechanisms to the correct patient populations. An important corollary of this conclusion is that patients will be the ultimate beneficiaries of more concerted collaboration between academic and industrial laboratories since the former possess cutting edge genetic resources and specialized methodology, which can be paired with the cutting edge pharmacological tools developed by the latter.

## The Next Step: A Cross-Species Translational Push and Conceptual Framework

To develop effective therapies and conduct successful translational research, we must have a reasonable grasp of human neural phenotypes reflecting brain dysfunction as well as the brain circuitry underlying such phenotypes. Next, we can seek to develop therapeutic approaches (e.g., with small molecule drugs, targeted deep brain stimulation (DBS), and behavioral therapy) that are effectively targeted to modulate a given set of phenotypes. For instance, pioneering studies by Wilder Penfield and Theodore Rasmussen began to connect medial temporal lobe functionality with episodic memory (Penfield and Rasmussen, 1950); the study of H.M. (Henry Molaison) by William Scoville and Brenda Milner further implicated the hippocampus and adjacent cortices in episodic memory (Scoville and Milner, 1957); volumetric and neuroimaging studies in human patients with major depression have pointed toward key temporal lobe areas involved in the pathophysiology of depression (Baxter et al., 1989; Ongür et al., 1998; Mayberg et al., 1999; Rajkowska et al., 1999; Pizzagalli et al., 2005; Epstein et al., 2006; Siegle et al., 2007; Gittins and Harrison, 2011); atrophy and functional deficiency of the hippocampus, entorhinal cortex and fornix point toward a key role of the entorhinal-hippocampal system in AD (Small et al., 2011; Jagust, 2013; Khan et al., 2014); high resolution fMRI studies in aMCI patients point toward hippocampal subfield-specific functional deficits and pathology (Yassa and Stark, 2011; Bakker et al., 2012; Leal and Yassa, 2016); neuroimaging studies in PTSD patients have shown amygdala hyperactivation (Rauch et al., 2000; Shin et al., 2004; Vermetten et al., 2007), prefrontal cortex hypoactivation (Bremner et al., 1999a, 2005; Shin et al., 1999, 2001, 2005; Lanius et al., 2003; Britton et al., 2005; Phan et al., 2006; Kim et al., 2008) and hippocampal hypoactivation (Bremner et al., 2003; Shin et al., 2004; Vermetten et al., 2007).

The substantial amount of extant human clinical data provide sizeable platforms for reverse translating these findings to animal models for causal and mechanistic interrogations. For instance, extensive work has demonstrated that aging biases people to rely more on gist-based memory and previously encoded information (Devitt and Schacter, 2016). As a cellular correlate, DG neurogenesis declines with age and compromises the ability of the hippocampus to engage in mnemonic discrimination in the service of forming novel orthogonal engrams (Denny et al., 2014; Danielson et al., 2016). Compounding this decrement, work in both rats and rhesus macaques has shown that CA3 pyramidal cell firing rates become significantly elevated and correlate with mnemonic discrimination impairments (Wilson et al., 2005, 2006; Thomé et al., 2016). Indeed, recent work has shown that hippocampal processing in older adults, early aMCI patients and age-impaired animals is shifted toward pattern completion (Wilson et al., 2005, 2006; Yassa and Stark, 2011; Yassa et al., 2011; Bakker et al., 2012; Stark et al., 2013; Leal and Yassa, 2016). BOLD fMRI work has revealed DG/CA3 hyperactivity in early aMCI patients that correlates with a compromised ability to effectively discriminate similar lure objects (Yassa and Stark, 2011; Yassa et al., 2011; Bakker et al., 2012; Stark et al., 2013; Leal and Yassa, 2016). Moreover, studies in age-impaired rodents and macaques have demonstrated a decrease in GABAergic inhibitory interneuron markers in the DG hilus and CA3 that serve as a cellular correlate of this age-related disinhibition of hippocampal network activity (Spiegel et al., 2013; Thomé et al., 2016). Following continued disease progression and neurodegeneration, the hippocampal hyperactivity in early aMCI gives way to hippocampal hypoactivity in late aMCI through AD (O’Brien et al., 2010). Taken together with the convergent findings from the animal literature this suggests that CA3 neuronal hyperactivity causes the early aMCI hippocampus to become biased toward representing previously coded information (pattern completion) at the expense of being able to encode novel information (pattern separation).

We return to the issue of two M_1_ activators eliciting opposing effects on hippocampal processing and judge the therapeutic implications through our proposed cross-species framework. The authors found that the M_1_ PAM and M_1_ agonist biased hippocampal processing toward either pattern separation or pattern completion, respectively (Lebois et al., 2016). Which mechanism could be leveraged to treat episodic memory impairments in an early aMCI population or an AD population? Since aMCI patients and age-impaired animals exhibit markedly elevated CA3 activity that corresponds to excessive pattern completion (and by extension, impaired pattern separation), a drug which diminishes pattern completion by CA3 and/or bolsters pattern separation by DG would likely be beneficial for normalizing episodic memory circuitry function in early aMCI. Although M_1_ PAMs and agonists both improve memory in the Morris Water maze, the observation that M_1_ PAMs shift hippocampal processing toward pattern separation suggests that this mechanism may improve episodic memory in early aMCI patients, whereas an M_1_ agonist may further exacerbate their memory dysfunction. However, with the significant degeneration and loss of excess pattern completion in late aMCI and AD (O’Brien et al., 2010), an M_1_ agonist may be needed to overcome the loss of acetylcholine tone and properly normalize hippocampal network function.

## A Convergence of Stimulation Strategies

A second cross-species case study that bears examining is the juxtaposition of the human DBS literature with rodent memory reactivation.

Pioneering work by Mayberg et al. (2005) has led to the subgenual cingulate (SCC) as a candidate DBS target for MDD patients who suffer from treatment-resistant depression. Importantly, at 1, 2 and 3 year follow-ups, the average response rates (defined as a decrease of ≥50% in total score on the 17-item Hamilton Depression Rating Scale [HAM-D]; Hamilton, 1960) for MDD patients receiving DBS remained 62.5%, 46.2% and 75%, respectively, indicating that DBS can exert long-lasting safe and beneficial effects (Kennedy et al., 2011). Additional brain areas have been targeted in DBS case studies including the ventral capsule/ventral striatum, NAcc and medial forebrain bundle (Crowell et al., 2014). A tremendous amount of structural MR work and task-related BOLD fMRI work in affected MDD patients has led to the targeting of all of the above candidate regions. Volumetric alterations have been demonstrated in the SCC hippocampus, and dorsolateral prefrontal cortex (DLPFC; Ongür et al., 1998; Rajkowska et al., 1999; Gittins and Harrison, 2011). Based upon an equally large body of task-related BOLD fMRI studies, hypermetabolic activity has been observed in the SCC (BA25), while hypometabolic activity has been observed in the DLPFC, ventral striatum and NAcc (Baxter et al., 1989; Mayberg et al., 1999; Pizzagalli et al., 2005; Epstein et al., 2006; Siegle et al., 2007). In support of the hypothesis that BA25 is involved in negative mood regulation, a decrease in BA25 activity has been consistently observed in acute sadness and in response to antidepressant treatment strategies including SSRI dosing, TMS, ECT and surgical ablation (Malizia, 1997; Mayberg et al., 1999, 2000; Nobler et al., 2001; Dougherty et al., 2003; Goldapple et al., 2004; Seminowicz et al., 2004).

A related body of work has been developing in the rodent engram literature to genetically tag and modulate memories (Ramirez et al., 2015). The authors found that acutely and chronically reactivating positive, but not neutral or negative, memories reversed stress-induced neuronal and behavioral impairments. Additionally, positive memory reactivation elicited robust activity in a number of brain areas including the NAcc shell, lateral septum, basolateral amygdala, central amygdala, as well as dorsomedial, ventromedial and lateral hypothalamus. Moreover, inhibition of amygdala terminals in the NAcc was found to block the effects of positive memory reactivation.

The convergence of these two bodies of work sets the stage for the investigation of several outstanding questions in psychiatric disorders. Based on human literature, mice could be subjected to chronic stimulation protocols equivalent to what would be administered to human patients and to determine how they are altering the fidelity of memory expression and the pathways that may be disrupted or modulated (Friedman et al., 2014; Bagot et al., 2015; Creed et al., 2015). Brain-wide cell activation patterns from animals can be compared to the aforementioned human imaging data to inform researchers on what brain areas might provide antidepressant efficacy and serve as targets for DBS (Ressler and Mayberg, 2007).

## Fear Memory Reactivation and Exposure Therapy for PTSD

A final cross-species case study that is important to examine is the convergence of the human PTSD literature with fear memory modulation studies from the rodent literature. PTSD is a disorder marked by overgeneralization of a learned fear response to situations that would ordinarily be considered safe, often resulting in a state of autonomic hyperarousal (Mahan and Ressler, 2012). A wealth of neuroimaging studies have helped to define the neural circuitry and intermediate phenotypes associated with PTSD, as patients typically display amygdala hyperactivity (Rauch et al., 2000; Shin et al., 2004; Vermetten et al., 2007), hippocampal hypoactivity (Bremner et al., 2003; Shin et al., 2004; Vermetten et al., 2007), prefrontal hypoactivity (Bremner et al., 1999b, 2005; Shin et al., 1999, 2001, 2005; Lanius et al., 2003; Britton et al., 2005; Phan et al., 2006; Kim et al., 2008) and anterior cingulate hyperactivity (Shin et al., 2001, 2006; Bryant et al., 2005). Behaviorally, patients with PTSD are known to display marked deficits in fear extinction learning (Orr et al., 2000; Milad et al., 2008).

**Figure 2:**
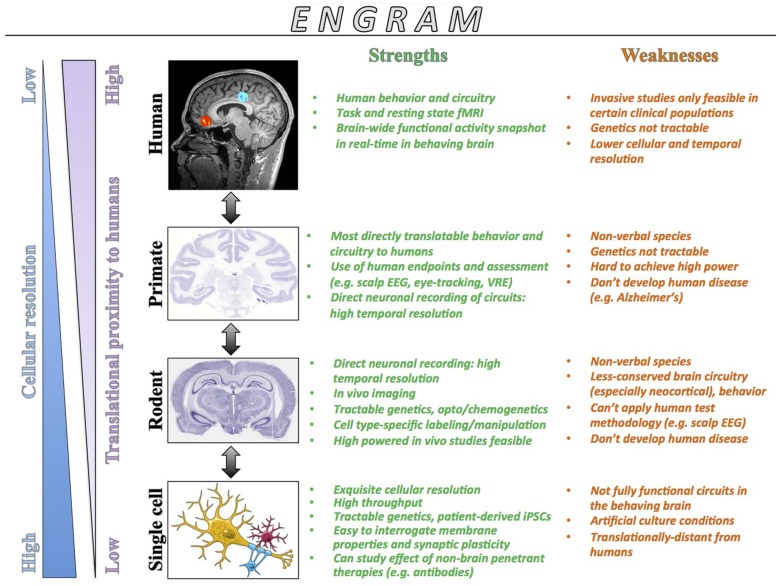
**A top-down cross-species approach to memory and psychiatric disorders.** Research at multiple levels of analysis and resolution carry with it its own unique set of experimental strengths and weaknesses described above. Note that this list is not intended to be exhaustive, but rather representative of the major strengths and weaknesses of each system. In this respect, a top-down back-translational approach anchored around established human findings and methodology provides concrete directionality for questions posed in animals and cells. Given the seemingly infinite number of manipulations that can be made at the animal and cellular level, this approach helps to both parameterize this vast space and ensure the translational relevance of animal and cellular findings. Three particular junctures are especially noteworthy. First, work in nonhuman primates represents the convergence of behavior and brain structure that most closely approximates that of the human. Essentially, human methodology can be applied to measure the same endpoints in the same way and paired with techniques to directly record neuronal circuit activity in behaving animals. Second, rodents represent the convergence of tractable genetics with similar neuronal recording capabilities of nonhuman primates. Finally, work at the single cell level allows us to easily interrogate functional membrane properties, synaptic plasticity, and also work with patient-derived stem cells.

A broad body of work from Ressler et al. (2004) serves as an example *par excellence* of how human genetics might be leveraged to help us better stratify patients, understand human disease biology, and build in more translational relevance to preclinical approaches. In particular, the use of candidate gene approaches has led to the identification of polymorphisms associated with PTSD risk in FKBP5, ADCYAP1R1, SERT, COMT, BDNF, GABRA2 and ApoE2 (reviewed in Lebois et al., 2016). For example, a sex-specific single nucleotide polymorphism (SNP), rs2267735, in an estrogen response element within the PAC1 receptor gene (ADCYAP1R1) was found to be predictive of PTSD diagnosis and symptoms in females (Ressler et al., 2011). The identification of such genetic polymorphisms when paired with studies aimed at studying neural intermediate phenotypes that render patients susceptible to, or are associated with recovery from, trauma symptoms holds tremendous promise. Moreover, such work dovetails with rodent research in which aversive memories have been ablated or temporarily inhibited by utilizing transgenic and optogenetic approaches (Han et al., 2009; Denny et al., 2014). Indeed, a strong foundation for the next generation of clinical studies can be enabled by facilitating better patient stratification as well as by targeted back-translational approaches aimed at gaining a mechanistic understanding of fear memories (Figure 2) as well as the underlying anatomy supporting fear-relate responses.

## The NIH’s Research Domain Criteria (RDoC)

Research Domain Criteria (RDoC) is a framework compiled by NIH in attempt to move away from the descriptive and observational nosology of the Diagnostic and Statistical Manual of Mental Disorders (DSM) in favor a system that is grounded in brain circuits and functional domains (Cuthbert, 2014, 2015; Lillienfeld, 2014; Casey et al., 2015). A major driving factor in developing the RDoC framework was the desire to adopt a system that could link brain circuits directly with brain function in the service of enabling scientists to more directly pose and test neuroscience-based hypotheses. While the DSM has received much criticism because its system of descriptive and observational nosology does not lend itself to being concretely linked to the circuitry of the brain, the RDoC framework has suffered from criticism in many ways opposite to that of the DSM (Lillienfeld, 2014; Casey et al., 2015). Namely, it specifies empirically derived domains that are clearly tied to brain circuits, but do not clearly link to disease symptomology as defined by the DSM.

**Figure 3:**
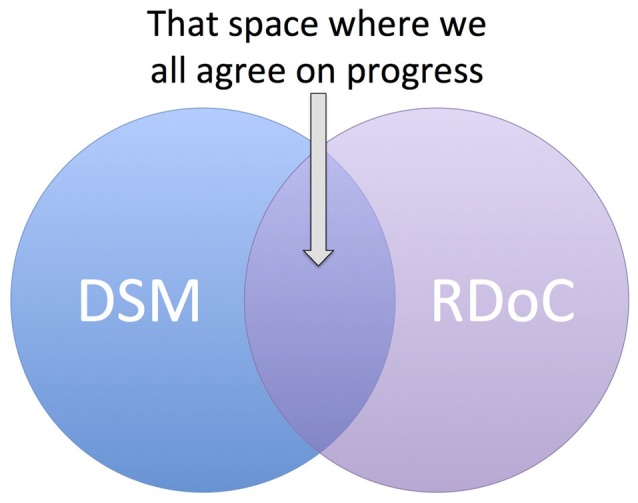
**Better together: the practical utility lies somewhere in between.** The Diagnostic and Statistical Manual of Mental Disorders (DSM) has received much criticism because its system of descriptive diagnostic nosology does not lend itself to being concretely linked to the circuitry of the brain, which renders the DSM difficult to use as a construct that motivates hypothesis-driven neuroscience research. The Research Domain Criteria (RDoC) framework has suffered from the opposite problem. Namely, it specifies empirically derived domains that are clearly tied to brain circuits, but do not clearly link to disease symptomology as defined by the DSM. Particularly as intermediate phenotypes are better defined in human patient populations and reconciled with circuit-level observations from animal studies, the functionalization of the RDoCs framework can occur and be more accurately mapped onto the symptom-driven DSM nosology to achieve a middle ground that is practically useful by both physicians and researchers.

A cross-species framework, similar to a cross-species study of memory, could enable the RDoC and DSM camps to meet on a middle ground of functional utility (Figure 3). This sort of cross-species approach to engram research fits within the existing RDoC structure can be extended in multiple ways. It adds a causal understanding of memory processes that can be linked to the domains of cognitive systems. By being anchored around human functional neuroscience, genetics, and the endophenotyping of human clinical populations, the conceptual advance of our proposed framework is a call to arms to begin earnestly linking disease symptomology to psychiatric and neurologic endophenotypes of disease and the underlying affected neural circuitry.

## Conclusions

In the last decade, neuroscience has witnessed an explosion of technological and conceptual breakthroughs that have made it possible to visualize and manipulate the neural correlates of memory with exquisite resolution and precision. We are as optimistic about the modern state of memory research as we are realistic about the amount of science that remains to be performed before we understand the principles organizing memory. For example, any logic underpinning memory must also take into consideration the dynamic nature of memory across circuits and across time. The progress is already underway: it was recently demonstrated that the paraventricular nucleus of the thalamus acts as a crucial nexus for differentially recruiting cortico-amygdalar networks as a memory consolidates over weeks (reviewed in Do Monte et al., 2016). The level of sophistication in experiments manipulating internally generated representations is growing as well. For example, a tremendous advance includes newly developed two-photon and optogenetic methods for artificially imprinting activity onto neuronal ensembles such that single-cell stimulation leads to retrieval, or completion of, the original pattern of ensemble activity (Carrillo-Reid et al., 2016). Another example includes recent closed loop experiments, which have demonstrated that pairing place cell activity to rewarding stimulation of the medial forebrain bundle during wakefulness or sleep is sufficient to create a place preference (de Lavilléon et al., 2015), thus opening up the possibility of utilizing similar closed loop paradigms to artificially associate, enhance, or inhibit engrams in a psychiatric disease-related setting as well. Indeed, the combination of genetically defined and cell-type specific techniques will not only yield causal insight into memory’s neural substrates but will also enable the rational design for interventions capable of reprogramming the brain in a therapeutic manner (Creed et al., 2015).

Still, many questions abound: what are the endogenous firing properties of engram cells tagged during learning (e.g., are they place cells, time cells)? How do the physiological firing properties of these cells change for recent and remote memories, or for memory updating and extinction, across an array of circuits, both in terms of cell bodies and projections? How does the transcriptional and translational landscape of engram cells change in response to the various phases of memory? What role do neuromodulators play in changing the molecular and physiological properties of engram cells (Marcinkiewcz et al., 2016)? What are the underlying principles enriching cortico-hippocampus engram cells with the capacity to process both space and time (Eichenbaum and Cohen, 2014)?

As the engram literature bourgeons, every new finding requires us, as a community of memory researchers, to follow the rules of engagement: respectful, constructive and curiosity-driven dialogs that enable us to stand on each other’s shoulders and not on each other’s feet. Indeed, we are confident that the next decade of neuroscience will usher in a new era of memory research; after 100 years of remaining dormant, the elusive engram has at last reawakened.

## Author Contributions

CAD, EL and SR all wrote the article.

## Conflict of Interest Statement

The authors declare that the research was conducted in the absence of any commercial or financial relationships that could be construed as a potential conflict of interest.
